# A Facile One-Pot Synthesis of Versatile PEGylated Platinum Nanoflowers and Their Application in Radiation Therapy

**DOI:** 10.3390/ijms21051619

**Published:** 2020-02-27

**Authors:** Xiaomin Yang, Daniela Salado-Leza, Erika Porcel, César R. González-Vargas, Farah Savina, Diana Dragoe, Hynd Remita, Sandrine Lacombe

**Affiliations:** 1 Institut des Sciences Moléculaires d’Orsay, CNRS, Université Paris-Saclay, 91405 Orsay, France; xiaomin.yang@universite-paris-saclay.fr (X.Y.); daniela.salado@conacyt.mx (D.S.-L.); farah.savina@universite-paris-saclay.fr (F.S.); 2 Facultad de Ciencias Químicas, Cátedras CONACyT, Universidad Autónoma de San Luis Potosí, Av. Dr. Manuel Nava 6, Zona Universitaria, San Luis Potosí 78210, S.L.P., Mexico; 3 Institut de Chimie Moléculaire et des Matériaux d’Orsay, CNRS, Université Paris-Saclay, 91405 Orsay, France; diana.dragoe@u-psud.fr; 4 Institut de Chimie Physique, CNRS, Université Paris-Saclay, UMR 8000, 91405 Orsay, France; hynd.remita@universite-paris-saclay.fr

**Keywords:** radiation, nanoparticle, platinum, radiolysis, radioenhancement, radiosensitization, cancer treatment

## Abstract

Nanomedicine has stepped into the spotlight of radiation therapy over the last two decades. Nanoparticles (NPs), especially metallic NPs, can potentiate radiotherapy by specific accumulation into tumors, thus enhancing the efficacy while alleviating the toxicity of radiotherapy. Water radiolysis is a simple, fast and environmentally-friendly method to prepare highly controllable metallic nanoparticles in large scale. In this study, we used this method to prepare biocompatible PEGylated (with Poly(Ethylene Glycol) diamine) platinum nanoflowers (Pt NFs). These nanoagents provide unique surface chemistry, which allows functionalization with various molecules such as fluorescent markers, drugs or radionuclides. The Pt NFs were produced with a controlled aggregation of small Pt subunits through a combination of grafted polymers and radiation-induced polymer cross-linking. Confocal microscopy and fluorescence lifetime imaging microscopy revealed that Pt NFs were localized in the cytoplasm of cervical cancer cells (HeLa) but not in the nucleus. Clonogenic assays revealed that Pt NFs amplify the gamma rays induced killing of HeLa cells with a sensitizing enhancement ratio (SER) of 23%, thus making them promising candidates for future cancer radiation therapy. Furthermore, the efficiency of Pt NFs to induce nanoscopic biomolecular damage by interacting with gamma rays, was evaluated using plasmids as molecular probe. These findings show that the Pt NFs are efficient nano-radio-enhancers. Finally, these NFs could be used to improve not only the performances of radiation therapy treatments but also drug delivery and/or diagnosis when functionalized with various molecules.

## 1. Introduction

Over the past decades, there has been increasing interest in the use of formulations to enhance radiotherapeutic effects, especially using metal (high Z atom) based nanoparticles (NPs) [1]. The introduction of high-*Z* NPs into a tumor offers the possibility of amplifying radiation damage effects selectively in the tumor as compared to healthy tissues, thus enhancing therapeutic efficacy [2]. Gold NPs (Au NPs) are the most studied nano-radio-enhancers (NREs), owing to their high electronic density in particular, which is favorable to amplification of radiation effects. A pioneering study by Hainfeld et al. demonstrated that small Au NPs (1.9 nm in diameter) prolong the life of mice treated with 160 kV X-rays [3,4]. Thereafter, other studies, using in vitro assays as well as xenografts, focused on the enhancing effect of Au NPs in radiation therapy using X-rays, γ-rays, proton, and electron beams etc [5,6]. Gadolinium (Gd) was identified as another valuable theranostic enhancer for magnetic resonance guided radiation therapy. Recently, the sub-5 nm Gd NP (AGuIX, developed by NH TherAguix, Grenoble, France) consisting of a Gd oxide core surrounded by a polysiloxane shell and grafted with diethylenetriaminepentaacetic acid (DTPA) was found to possess efficient in vitro radiosensitizing properties at a photon energy of 660 keV [7,8]. Nanoparticles containing other heavy elements, for example, 50-nm crystalline HfO2 NPs (NBTXR3, Nanobiotix, Paris, France), were also developed. It has been demonstrated that these NPs exposed to high-energy photons induce a significant radiation dose enhancement as shown in vitro and in vivo [9,10,11].

Relatively few studies have investigated the radio-enhancing and synergistic effects of Pt NPs for ionizing radiation. Le Sech et al. and Kobayashi et al. reported that chloroterpyridine platinum (PtTC) bound to plasmid DNA could enhance the X-ray-induced breaks in DNA plasmid in an aqueous solution [12,13]. However, these compounds are nonselective for tumor cells. Hence, Pt-based NPs were developed for cancer treatment exploiting the enhanced permeation and retention (EPR) effect. Porcel et al. presented the prominent radio-enhancement of Pt NPs at a Pt concentration of 5.6 × 10^−6^ mol L^−1^ (~1 µg/mL), which augments the breaks in DNA with X-rays, γ-rays and fast ions [14,15]. Their results demonstrated that the production of water radicals is mainly involved in the effect of NPs to amplify radiation-induced damages (indirect process), while the direct process (including direct breaks induced by photons or incident particles) contribute less, as also indicated by Kim et al. [16] and others [17,18].

Cancer radiation therapy is closely related to the capability of cellular uptake of NPs, which can be determined by its surface chemistry and particle size [19]. So, it is essential to prepare small size, biocompatible, nontoxic NPs. The synthesis of Pt NPs has already been studied using different methods such as chemical reduction [20], light assisted reduction by γ-rays radiation [21], UV radiation [22], and biologically assisted procedures [23]. Among these techniques, radiolysis is considered as an effective method for the synthesis of metallic NPs [24]. Solvated electrons of very high reducing power and reducing radicals are generated by solvent radiolysis. This method has the advantage of homogeneous reduction and nucleation leading to metallic nuclei in water with 100% yield and a narrow particle size distribution without subsequent steps of purification due to the lack of pollutants, providing a green alternative over chemical methods. Stabilizers, such as dextran [25], polyacrylic acid (PAA) [26], chitosan [27], and others, have been used for the protection of Pt NPs from agglomeration. Among the stabilizers studied, PEG (Poly(Ethylene Glycol)) is one of the versatile and eco-friendly polymers that is particularly interesting in the synthesis of metallic NPs due to its interactions with metallic NPs through both steric and electrostatic effects [28]. Furthermore, coating NP surfaces with PEG chains is one of the best modifications to increase the EPR effect because the coating prolongs the circulation time of NPs in the blood [29]. As previously mentioned, the efficacy of the metal-based formulation depends upon the energy of the radiation along with the type, amount and location of material within the tissue. Better targeting and pharmacokinetic profile of the NPs will generate much more efficient therapy with reduced adverse effects to surrounding healthy tissue [30,31].

To the best of our knowledge, there has been no research on the synthesis of platinum nanoflowers (Pt NFs) with small and uniform size by the radiolytic method. NFs are a special category of nanoscale materials containing a special combination of elements which, when viewed microscopically, look like flowers [32,33]. Nowadays, NFs have attracted intensive attention due to their high resistance, simple preparation processes, increased efficiency, and high stability [34]. In this study, the synthesis of Pt NFs by radiolysis (with Cobalt-60 γ-rays) using PEG-diamine as a stabilizer was carried out, and the physico-chemical properties were characterized. Furthermore, we investigated their radiation dose enhancement potential in vitro on cervical cancer (HeLa) cell line and explored the mechanisms driving the cellular response to photon radiation.

## 2. Results and Discussions

### 2.1. Synthesis and Characterization of Pt NFs

Water radiolysis is a convenient and environmentally friendly approach to synthesize monodisperse metallic NPs at a large scale as it generates in the entire solution uniformly distributed reducing species (solvated electrons and H^•^) that reduce metallic ions (Pt^2+^) during irradiation [35,36]. The radiolytic production of PEGylated metallic NPs for biomedical applications has been recently patented: Nanoparticules et procédé de preparation, FR1900008.

**Figure 1:**
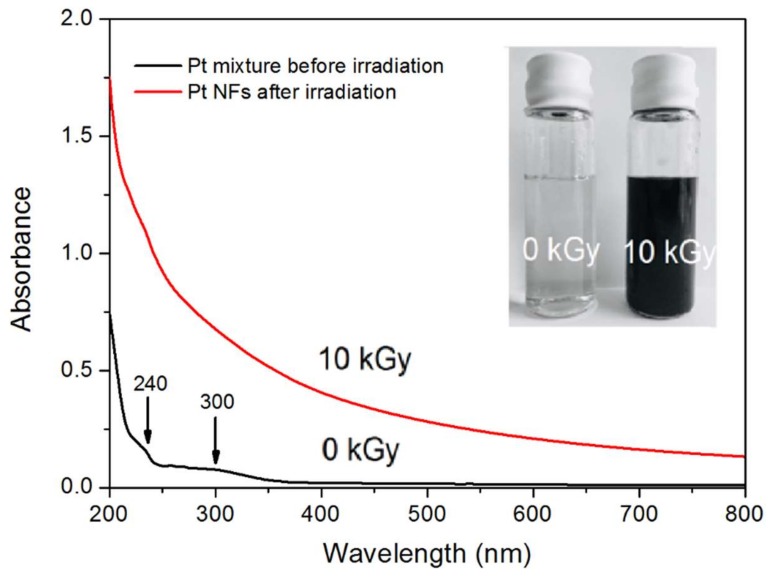
UV-Vis absorption spectra of diluted Pt containing solutions (10^−3^ mol L−1) before (black line) and after (red line) irradiation (optical path = 2 mm). Inserts: images of solutions resulting from 0 and 10 kGy radiation doses.

The reduction of Pt^2+^ ions into Pt^0^ and the formation of Pt NPs was monitored using UV-vis absorption spectroscopy. Figure 1 shows the UV-vis spectra of an aqueous solution containing the precursor (Pt(NH_3_)_4_Cl_2_) and PEG-diamine, at a 1:50 molar ratio, before and after γ-ray irradiation. The inserted picture displays the solution before (left) and after irradiation (right). The initial (before irradiation) colorless solution turns dark brown after irradiation. It is a first indication of the formation of Pt NPs [37].

In addition, the UV-Vis spectrum obtained before irradiation exhibits an absorption band around 300 nm with a weak band at 240 nm. These peaks are attributed to the ligand-to-metal charge transfer (LMCT) of the Pt precursor [37]. After irradiation, the appearance of the stretched absorption spectrum with significant increase in intensity, suggests the formation of Pt NPs [38]. The same pattern of the UV-Vis spectrum of Pt NPs with no maximum absorbance was reported by Zhang et al [39] using the citrate reduction method, and Remita et al. [21], Choi et al. [40] and Nguyen et al. [27] using radiolysis. However, a surface plasmon resonance peak of Pt NPs in wavelength of 215 nm was observed by Cele et al. [41] and Gharibshahi et al. [42] through the γ-ray irradiation method. The changes in resonance peak depend on the particle shape, size and the method that is used for the synthesis of NPs [43].

**Figure 2:**
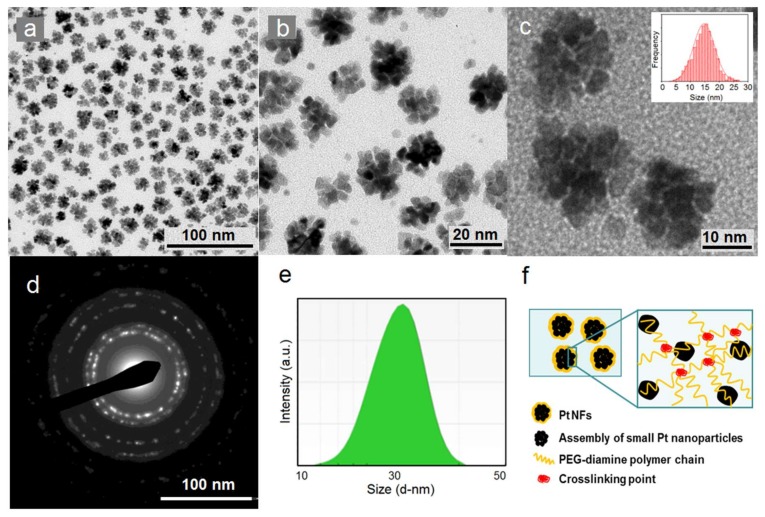
(**a**–**c**) Lower and higher magnification High-Resolution Transmission Electron Microscopy (HR-TEM) images of platinum nanoflowers (Pt NFs) and HR-TEM size distribution histogram of Pt NFs (inset); (**d**) Electron diffraction pattern of Pt NFs; (**e**) Dynamic Light Scattering (DLS) size distribution by intensity of Pt NFs; (**f**) Scheme of the formation of Pt NFs by radiation-induced polymer cross-linking.

High-Resolution Transmission Electron Microscopy (HR-TEM) images are presented in Figure 2a–c. They allow determining the size distribution and morphology of the synthesized Pt NPs. As shown in Figure 2a, uniform Pt NPs were obtained. Interestingly, NPs have flower-like shapes (Figure 2b). These “nanoflowers” (NFs) have a homogeneous size distribution with an average metallic core diameter of 14.6 ± 7.4 nm (inset in Figure 2c). From higher magnification (Figure 2c), we observe that these NFs appear to be self-assemblies of smaller objects (3.2 ± 1.6 nm) resulting from an aggregation mechanism [44]. The electron diffraction pattern of Pt NFs, shown in Figure 2d, indicated the good crystalline nature of the NFs. In the solution, as indicated in Figure 1e, the hydrodynamic diameter of the as-synthesized Pt NFs was about 34.8 ± 5.3 nm in water, well above the HR-TEM size, indicating the presence of PEG and hydration shell [45]. The zeta potential value at pH = 7 was measured to be 20 ± 2 mV. It was reported that nanoparticles with surface charge corresponding to a potential less than 20 mV can provide sufficient stabilization when using large molecular weight stabilizers (PEG-diamine), which act mainly by steric stabilization [46]. Moreover, to investigate the stability of Pt NFs in aqueous solution with different pH values (3–11), the sizes and zeta potential of Pt NFs were studied (see supporting information Figure S1). The hydrodynamic diameter of Pt NFs remained similar as the pH changes, with no visible precipitation.

The possible reaction and the evolution of Pt NFs were proposed as follows. First, the formation of the small Pt NPs from Pt ion solution by the γ-ray irradiation as described in detail in the patent (FR1900008). Briefly, Pt^II^ complexes were reduced to Pt0 mainly by hydrated electrons ($E^{\circ }\left(H_{2}O\;/{\;e}_{\mathrm{aq}}^{-}\right)=\;-2.9\;V$) and hydrogen radicals ($E^{\circ }\left(H^{+}/{\;H}^{\cdot }\right)=\;-2.3\;V$). The uniform energy distribution in the irradiated medium leads to homogeneous nucleation throughout the solution, and then Pt^0^ progressively coalesces to growing clusters. Polymers containing functional groups (PEG-diamine) were acting as stabilizers and capping agents due to their high affinity for Pt. These polymers anchor on the surface of NPs and provide electrostatic repulsion and steric hindrance [47]. Moreover, polymers played an important role in formation of NFs. The proposed mechanism is shown in Figure 1f, the stacked aggregations of small Pt NPs build up the NFs through the radiation-induced molecular cross-linking of polymer chains. Primary radicals (mainly hydroxyl radicals) formed by water radiolysis can be transferred to the polymer by hydrogen abstraction to form different kinds of macroradicals [48]. In the unique case of poly(ethylene oxide) chains, all the hydrogen atoms in the polymer molecule are equivalent, therefore only one type of macroradical $\left(–O–{\mathrm{CH}}_{2}–\;\dot{C}H–O–{\mathrm{CH}}_{2}–{\mathrm{CH}}_{2}–\right)\;$was formed [49]. Macroradicals can be terminated by molecular crosslinking as these carbon centered radicals were generated simultaneously along each polymer chain in solutions [48].

The freshly prepared PEGylated NFs were stable in solution for one month at 4 °C. To ensure long-term storage, they were successfully lyophilized using a two-step freeze drying method. The lyophilized product preserved the physiochemical properties and can be stored for several months at room temperature. Re-suspension in various biocompatible buffers (such as PBS 10 mM, HEPES 1 M) do not modify the characteristics of the NFs.

**Figure 3:**
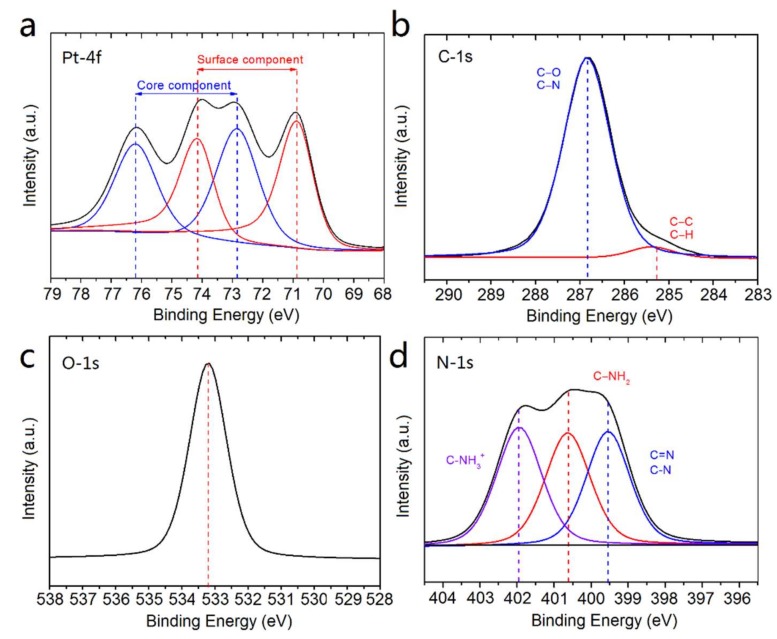
X-ray Photoelectron Spectroscopy (XPS) spectra of Pt NFs at (**a**) Pt-4f, (**b**) C-1s, (**c**) O-1s, (**d**) N-1s regions.

X-ray Photoelectron Spectroscopy (XPS) analysis was used to elucidate the chemical composition and to determine the oxidation state of Pt NFs. The XPS survey spectrum (see supporting information Figure S2) shows the presence of Pt, C, O, N and Cl signals of Pt NFs. The high-resolution XPS spectrum at the Pt-4f region is shown in Figure 3a. For the curve fitting analysis, a spin-orbit doublet binding energies (BEs) splitting of 3.3 eV was used. The spectrum can be deconvoluted into two doublets, one at 70.8 and 74.1 eV (in red) and the other one at 72.8 and 76.1 eV (in blue). These two BEs of each doublet correspond to the Pt-4f_7/2_ and Pt-4f_5/2_ spin-orbit components of Pt [50]. The low BEs correspond to the surface component of Pt^0^, while the high BEs are attributed to the core component [51]. The results confirmed the complete reduction of Pt^2+^ ions into Pt metal atoms. In comparison with bulk Pt, the BEs at the Pt-4f core level is shifted by about 1.5 eV, revealing bond formation between Pt and the PEG ligand [52]. Moreover, the full width at half maximum (FWHM) is a useful indicator of chemical state changes (see supporting information Table S1). The FWHM of Pt NFs at the Pt-4f core level, increased about 43%, from ~1.4 to 2.0 eV. This broadening of the peak confirms the interaction of Pt NFs with PEG molecules and indicating as well a flat PEG grafting [53].

In order to gain a better understanding of the chemical configuration of the PEGylated Pt NFs, the C-1s, O-1s and N-1s core levels were also investigated. The C-1s and O-1s spectra (Figure 3b,c) show the presence of the characteristic XPS signals of PEG chains around 286.8 and 533.2 eV, which correspond to C-O, C-N, C-O-C. It indicates that PEG-diamine preserve their native chemical structure after cross-linking. Remarkably, deconvolution of the N-1s spectrum shown in Figure 3d, reveals the presence of three components. The contribution at 402.0 eV is attributed to protonated alkylamine groups (C-NH_3_^+^), in which the C-NH_3_^+^ group may be interacting with the Pt NFs surface by electrostatic forces. This interaction between amine molecules and Pt atoms is well known in the literature [54,55,56]. The signal centered at 400.6 eV corresponds to free amine groups (NH_2_). The last component observed at 399.5 eV is attributed to C-N and C = N groups, the latter group suggests a possible grafting polymerization between the polymer macroradicals induced by radiation and the amine terminated groups of PEG-diamine [57].

These results of XPS analysis show that Pt NFs could be formed by chemisorption of PEG chains on metal surface through grafting polymerization and cross-linking.

**Figure 4:**
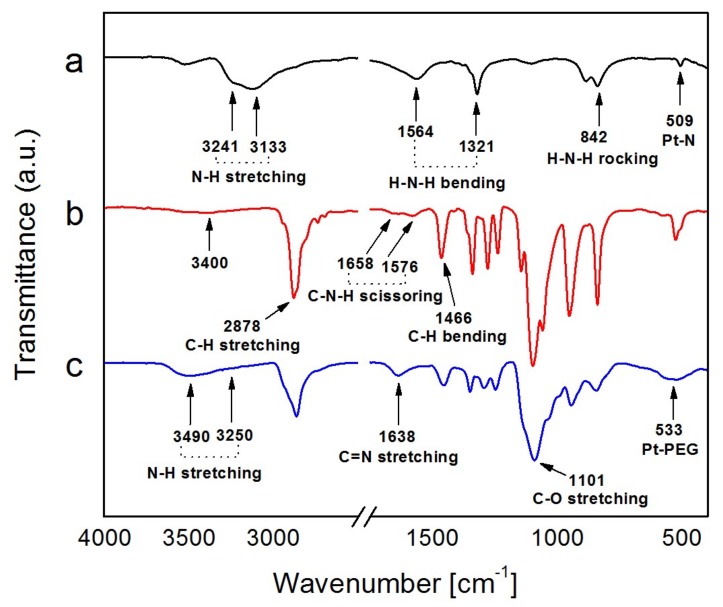
Fourier Transform InfraRed (FTIR) spectra of (**a**) Pt(NH_3_)_4_Cl_2_, (**b**) PEG-diamine 2000 and (**c**) Pt NFs.

Fourier Transform InfraRed (FTIR) spectroscopy was used to detect the binding of PEG-diamine on Pt NFs. Figure 4a shows the FTIR spectrum of the Pt precursor. We observed the amine symmetric and asymmetric stretching vibration at 3241 cm^−1^ and 3133 cm^−1^, in the region of 3000–3500 cm^−1^. The group of bands at 1564 cm^−1^, 1321 cm^−1^ is assigned to the -NH_2_ bending vibrational mode while the band at 842 cm^−1^ is ascribed to the NH_3_ rocking vibrational mode. The FTIR band at 509 cm^−1^ is attributed to the Pt-N stretching mode.

The spectrum of PEG-diamine (Mw = 2000 g mol^−1^) is shown in Figure 4b. The most striking feature is an intense band at 2878 cm^−1^ representing the stretching vibrations of the -CH_2_ group of polyethylene glycol [58]. The bands appearing at 1658 cm^−1^ and 1576 cm^−1^ are identified as the C-N-H scissoring mode of the amine terminal group of PEG. The rest of the bands are assigned as C–H bending at 1460 cm^−1^ and 1342 cm^−1^ and C–O stretching at 1101 cm^−1^. The very weak signal around 3400 cm^−1^ is attributed to the N-H stretching mode of primary amines.

The FTIR spectrum of Pt NFs is reported in Figure 4c, in comparison to PEG-diamine alone, the newly emerged band at 3250 cm^−1^ and 3490 cm^−1^ can be attributed to the symmetrical and asymmetrical N-H stretching mode of primary amine groups of the PEG-diamine ligand [59]. It indicates the amine groups are attached on the surface of Pt NFs. The most striking difference of this spectrum is the new band at 1638 cm^−1^ corresponding to the C = N stretching band for imines that occurs in the region from 1690 to 1620 cm^−1^. It supports the hypothesis of molecular crosslinking within the polymer molecules via radical interaction mechanism between the polymer radical $\left(–O–{\mathrm{CH}}_{2}–\;\dot{C}H–O–{\mathrm{CH}}_{2}–{\mathrm{CH}}_{2}–\right)$ and the amine group of PEG initiated by γ-ray irradiation. Moreover, the presence of a peak at 533 cm^−1^ confirms the functionalization on the Pt NFs surface with PEG-diamine. In summary, these results confirmed the successful integration of PEG-diamine to Pt NFs and the molecular cross-linking process is accompanied by a simultaneous grafting polymerization onto Pt NFs.

### 2.2. Toxicity of Pt NFs

**Figure 5:**
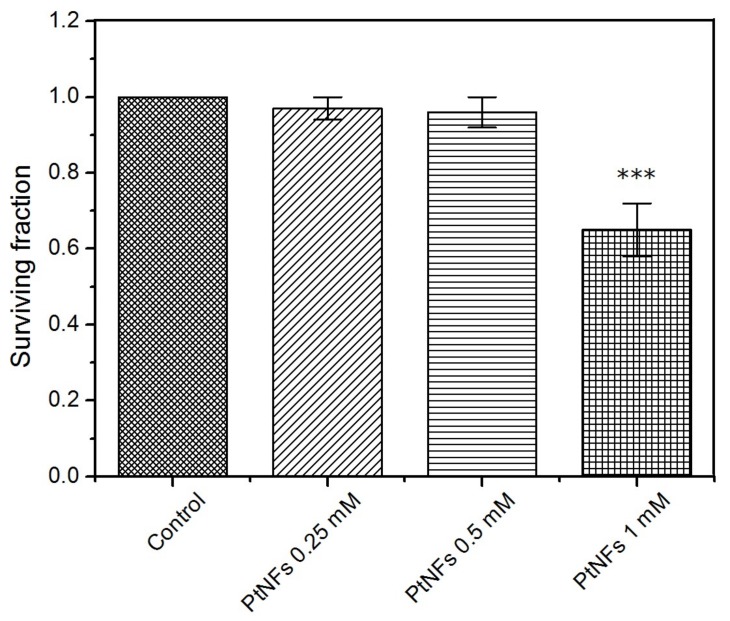
Cytotoxicity determined by clonogenic assay following a 6 h exposure to medium containing Pt NFs at different Pt concentrations of 2.5 × 10^−4^, 5 × 10^−4^ and 10^−3^ mol L^−1^. Data represent the mean ± SD of three identical experiments made in triplicate. Asterisks denote significant differences with control cells from ANOVA: *** *p* ˂ 0.001.

Colony-forming assay was used to determine the cytotoxic effects of Pt NFs and define the conditions of the irradiation study. As shown in Figure 5, no significant change in the Surviving Fraction (SF) was observed in HeLa cells following a 6 h exposure to 2.5 × 10^−4^ mol L^−1^ (49 µg/mL) or 5 × 10^−4^ mol L^−1^ (98 µg/mL) of Pt NFs, compared to the control group. However, the normalized SF reduced to around 65% when treated with 10^−3^ mol L^−1^ (196 µg/mL) of Pt NFs, showing cytotoxicity. Moreover, for all the concentrations, SF decreased to less than 60 % after 12 h incubation (see supporting information Figure S3). As such, a non-toxic consistent treatment concentration of 5 × 10^−4^ mol L^−1^ with 6 h incubation was selected for all subsequent experiments.

### 2.3. Fluorescent Labelling of Pt NFs

The Isothiocyanate derivative of Rhodamine B (RBITC) was chosen as fluorescent dye due to its suitable photophysical properties in water and good photo-stability [60].

**Figure 6:**
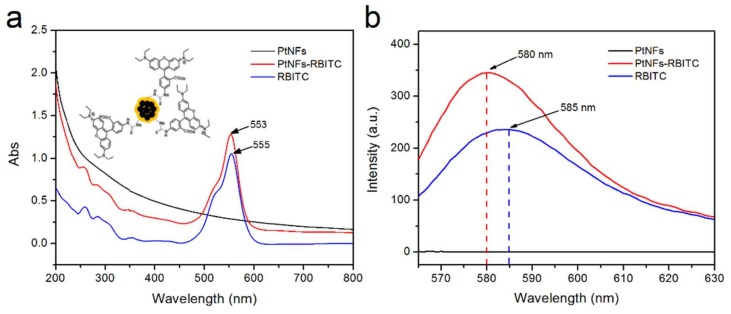
(**a**) UV-Vis absorbance spectra of Pt NFs (black spectrum), Rhodamine B Isothiocyanate (RBITC) labeled Pt NFs (Red spectrum), free RhB-ITC (blue spectrum). Insert shows the schematic illustration of RBITC labeled Pt NFs. (**b**) Fluorescence emission spectra (λexc = 555 nm) of Pt NFs, RBITC labeled Pt NFs, RBITC.

The successful covalent attachment of RBITC has been proved by UV-Vis spectroscopy. A 2 nm blue shift was observed in the absorption spectrum of RBITC labeled Pt NFs compared to the RBITC alone. As shown in Figure 6a, the absorption spectrum of RBITC features a typical band at 555 nm characteristic to the π-π* electronic transition in the xanthene ring [61]. Similar shifts of the absorbance peak were reported previously in other studies of Rhodamine conjugated to silver NPs or carbon dots (CD) [62,63]. Diac et al. indicated the conversion of the isothiocyanate moiety in RBITC molecule to the thiourea upon the reaction with the terminal primary amine in the CD-PEG [58]. The blue shift was ascribed to modification of the molecular structures as the angle between the xanthene plane and the phenyl ring changes by 4–6 degrees, as shown in the insert of Figure 6a. Therefore, we speculate the covalent attachment of the RBITC to Pt NFs will change the angle between the xanthene plane and phenyl substituent introducing variation in the maximum absorption of RBITC labeled Pt NFs.

Further, we investigated the fluorescence properties of RBITC in the presence of Pt NFs. The fluorescence intensity of RBITC was enhanced upon the addition of Pt NFs, as seen in Figure 6b. The enhanced fluorescence can be attributed to the proximity of the fluorophore with the metallic NPs, causing metal enhanced fluorescence (MEF) [64]. It is well known that MEF effect is strongly dependent on the distance between metal surface and fluorophore. Moreover, we notice the blue-shift of the RBITC emission from 585 nm to 580 nm as a strong evidence of the dye molecules attachment to the metallic surface of NFs.

### 2.4. Pt NFs Localization and Quantification

**Figure 7:**
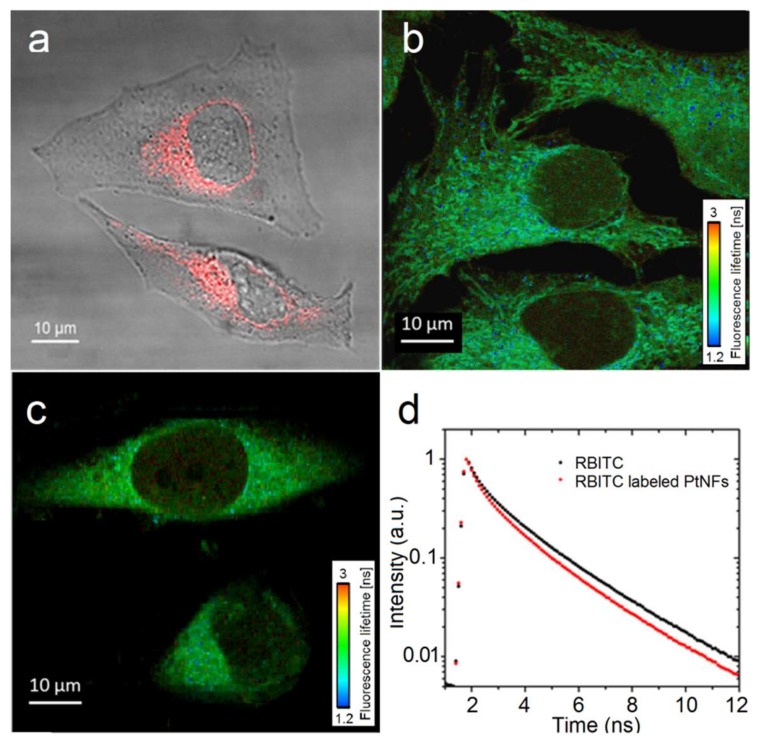
(**a**) Merged image of the transmission and fluorescence images obtained by confocal microscopy of HeLa cell loaded with RBITC labeled Pt NFs at a concentration of Pt of 5 × 10^−4^ mol L^−1^, incubated for 6 h; (**b**,**c**) The Fluorescence Lifetime Imaging Microscopy (FLIM) imaging of cervical cancer cells (HeLa) incubated with RBITC labeled Pt NFs and free RBITC respectively, fluorescence lifetime is showed in the nanosecond range; (**d**) Fluorescence decay curves (mean lifetime curve) of the free RBITC (black) and of the RBITC labeled Pt NFs (red) after an excitation at 550 nm.

Labelling Pt NFs with fluorescent dyes enables studies of intracellular localization of Pt NFs. Figure 7a shows confocal microscopy images of HeLa cells loaded with RBITC labeled Pt NFs. Interestingly, Pt NFs at a Pt concentration of 5 × 10^−4^ mol L^−1^ were localized in the cytoplasm of HeLa cells without penetrating into the nucleus. However it is still ambiguous whether the dye RBITC was still covalently bound to the Pt NFs when enter inside the cells. The Fluorescence Lifetime Imaging Microscopy (FLIM) measurements were performed to obtain a more accurate and detailed insight into the biophysical environment of the fluorophore.

The FLIM images of HeLa cells incubated 6 h with RBITC labeled Pt NFs or free RBITC are presented in Figure 7b,c. The fluorescence intensity found in the case of RBITC labeled Pt NFs is higher in comparison to the signal with free RBITC. In Figure 7d, the fluorescence lifetime (τ) curves of free RBITC and RBITC labeled Pt NFs show a clear lifetime decrease, from ~2.5 ns to ~2.3 ns (see supplementary Table S2 for detail). Strong effects on the fluorescence emission intensity and the fluorescence decay rates are expected when fluorophores are bound to metal surfaces, especially to metallic NPs [65]. The observation implicated a stable dye-tag and ensured that the signals observed in the HeLa cells after 6 h incubation are due to the fluorescent NFs. The location of the NPs in the cytoplasm near the nucleus is in agreement with the observation of Stefancikova et al. with Gd-based NPs. They highlight a significant cell killing enhancement of glioblastoma (U87) cells treated with Gd-base NPs prior to γ-rays radiation despite the location of the radio-enhancing agent in the cell cytoplasm [7]. In addition, Au NPs were found localized in the cytoplasm in breast-cancer cell line (MCF-7) and still provide noteworthy radio-enhancement under X-rays and γ-rays radiation [66].

Using Inductively Coupled Plasma Mass Spectrometry (ICP-MS), we quantified the uptake of Pt NFs in HeLa cells at an initial extracellular Pt concentration of 5 × 10^−4^ mol L^−1^. A mass of 0.462 μg of Pt was obtained from approximately 2.7 × 10^6^ cells, which corresponds to 0.171 pg of Pt per cell. Different results in uptake of radio-enhancing NPs have been observed in other studies depending on incubation time, concentration, cell line etc. For example, Li et al. described that approximately 0.0015 pg Pt are internalized in each D. radiodurans cell (prokaryote cells) after an overnight incubation with Pt NPs ([Pt] = 10^−3^ mol L^−1^) [67]. Furthermore, Luchette et al. shows that ~0.028 pg of Gd are taken up per HeLa cell after 1 h of incubation with Gd-based NPs ([Gd] = 5 × 10^−4^ mol L^−1^/79 µg/mL) [68]. However, more studies are necessary to determine the mechanisms of NPs uptake and its efficacy regarding the effects of NP type, incubation time, concentration and cell line, etc.

### 2.5. Cell Irradiation

**Figure 8:**
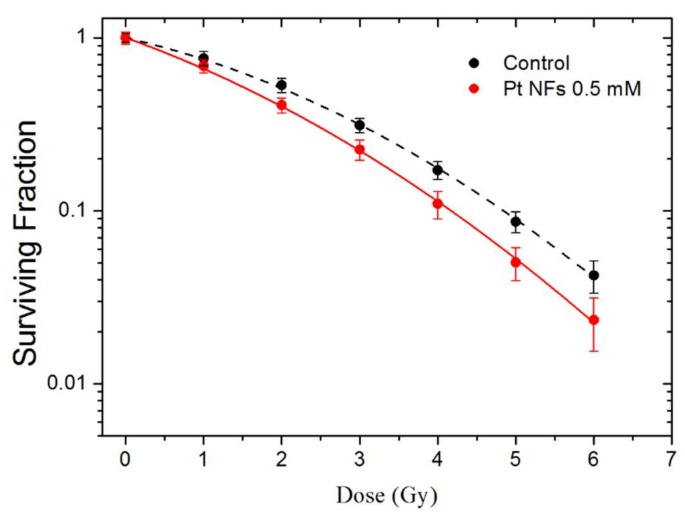
Surviving Fraction (SF) of HeLa cells irradiated by γ rays, in the presence of Pt NFs (red) and in the control (black).

The effect of Pt NFs on HeLa cells irradiated by γ-rays was investigated using clonogenic assay. The survival curves of HeLa cells free of Pt NFs (controls) and HeLa cells loaded with Pt NFs (at a Pt concentration of 5 × 10^−4^ mol L^−1^) irradiated by γ-rays are presented in Figure 8. The cell SF decreased while the radiation dose increased. This decrease was clearly amplified with in the presence of Pt NFs, indicating the radio-enhancement property of these Pt NFs.

The parameters α and β (see method section) of the linear quadratic fitting of survival curves are reported in Table 1. The Pt NFs induced an increase of the α parameter, from 0.24 to 0.37 while the β parameter remains nearly constant. The α parameter corresponds to the linear part of the curve its increase indicates an enhancement of the direct lethality of the radiation treatment in the presence of Pt NFs. The efficiency of Pt NFs to amplify radiation-induced cell death was quantified by the radiation sensitizing enhancement ratio (SER) and the dose enhancing factor (DEF). Previous studies have shown that the initial region of the survival curve at a dose of 2 Gy (Surviving Fraction at 2 Gy, SF^2Gy^) correlates well with clinical outcomes [69]. Therefore, SF^2Gy^ have been used to estimate the survival level at which to calculate SER_2Gy_ to investigate efficacy of NPs in vitro. SER_2Gy_ is defined as follows:

(1) $${\mathrm{SER}}_{2\mathrm{Gy}}=\;\frac{{\mathrm{SF}}_{\mathrm{control}}^{2\mathrm{Gy}}\;-{\;\mathrm{SF}}_{\mathrm{Pt}\;\mathrm{NFs}}^{2\mathrm{Gy}}}{{\mathrm{SF}}_{\mathrm{control}}^{2\mathrm{Gy}}\;}\;\times \;100\;\left[=\right]\;\%$$

${\mathrm{SF}}_{\mathrm{control}}^{2\mathrm{Gy}}\;$and ${\mathrm{SF}}_{\mathrm{Pt}\;\mathrm{NFs}}^{2\mathrm{Gy}}$ correspond to the SF obtained at 2 Gy, respectively for the control and for the cells loaded with Pt NFs. The ${\mathrm{SF}}_{\mathrm{control}}^{2\mathrm{Gy}}$ and ${\mathrm{SF}}_{\mathrm{Pt}\;\mathrm{NFs}}^{2\mathrm{Gy}}$ were 0.53 and 0.41, respectively. The SER_2Gy_ is close to 23%, which characterizes the Pt NFs efficiency when 0.66 MeV γ-rays are used as ionizing radiation (See Table 1). This is similar to a previous study reporting that Gd-based NPs at a gadolinium concentration of 5 × 10^−4^ mol L^−1^ efficiently amplify γ-rays induced cell killing of U87 glioblastoma cells by 23% under the same incubation conditions [7].

DEF is commonly used to assess the dose enhancement in vitro for a certain SF, commonly 10% [70].

(2) $$\mathrm{DEF}=\frac{D^{10}\mathrm{control}}{D^{10}\;\mathrm{Pt}\;\mathrm{NFs}}$$

where D^10^ corresponds to the dose at 10% of survival. The DEF of Pt NFs was found close to 1.20, this result means that the dose needed to kill 90% of the cell is divided by a factor of 1.20. It confirms that cell-killing effects are more pronounced by combining γ-rays with Pt NFs at a Pt concentration of 5 × 10^−4^ mol L^−1^. This is comparable to the effects with Au NPs at a concentration of 3 µg/mL after 24 h incubation, showing a DEF value of 1.10 using prostatic (PC3) cancer cell lines with X-ray irradiation [71]. Another study using PEG coated Au NPs (2 mg/mL for 24 h) in PC3 cells with X-rays radiation achieved a DEF of 1.15 [72]. These results suggest that Pt NFs could be an effective dose-enhancer for radiation therapies.

### 2.6. Nanosize Damage of Pt NFs

**Figure 9:**
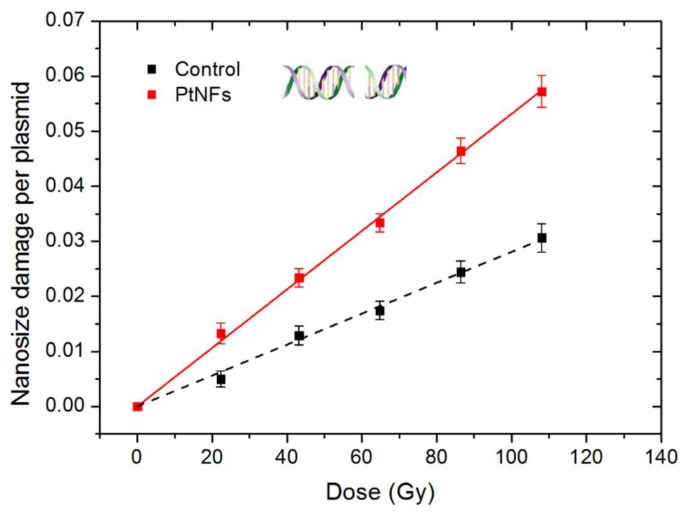
Nanosize damage induced by γ-rays in plasmids loaded with Pt NFs (red solid line) and in the control (black dash line) as functions of the irradiation dose.

In order to unveil the mechanism responsible for the radio-enhancement effects of Pt NFs, plasmids have been used as nano-bioprobes to detect and quantify the induction of bio-damage induced by early stage process (ESPs) (independent from cell metabolism). Since, complex damage in lipids, nucleic acids, proteins are the most lethal [73], we quantified here the induction of nanosize damage which is larger than 2 nm (distance between two strands), namely DSBs. The results obtained with plasmid loaded with Pt NFs and irradiated by γ-rays, are presented in Figure 9 as function of the irradiation dose from 0 up to 108 Gy. Pure DNA was used as control.

The radioinduction of nanosize damage in the control and in the DNA loaded with Pt NFs per plasmid increases linearly with the dose. When Pt NFs were added to DNA, the radiation effect was strongly amplified. This effect is in agreement with previous studies with Pt NPs coated with PAA and Gadolinium-based nanoparticles activated by photons, protons or heavy ions [15,74,75]. The efficiency of Pt NFs was quantified by calculating the molecular Amplification Factor (mAF):

(3) $$\mathrm{mAF}=\frac{{\mathrm{yield}}_{\mathrm{Pt}\;\mathrm{NFs}}-{\;\mathrm{yield}}_{\mathrm{control}}\;}{{\;\mathrm{yield}}_{\mathrm{control}}}\times 100\%$$

The yield is defined as the numbers of nanosize damage induced per plasmid and per Gray, which corresponds to the slope of the dose-response curves. This yield increases from 28.1 × 10^−5^ to 53.1 × 10^−5^ DSBs per plasmid and per Gy (See Table 2). The molecular amplification factor of Pt NFs corresponds to 89% of directly lethal damage amplification, higher than the reported value of Pt NPs combined with γ-rays which has 50% [15] directly amplification. We ascribed the amplification of these damages by Pt NPs as physico-chemical processes [2], starting with the enhancement of electron release in the medium compared to water due to atomic excitation by incident radiation. Subsequently, the excitation and ionization of water molecules close to NPs lead to the production of reactive radicals. When the diameter of NPs increases, we could expect a reduction of the electron release, because electrons coming from the bulk cannot escape the particle. In our case, the significant efficiency of Pt NFs at a Pt concentration of 4.23 × 10^−^^5^ mol/L (~8 µg/mL) is explained by the self-amplification of the electron emission that may take place between the subunits of a NF. Moreover, the path for the electrons to exit the metal remains similar as the path of the subunit. Thus, the production of radicals is amplified. Since these radicals are concentrated in nano-clusters, they induce complex nanosized bio-damages that are lethal for cells.

## 3. Conclusions

In this study, we demonstrated the efficient radiolytic method to produce, at large-scale, biocompatible Pt NFs with homogenous size distribution, capable to enhance radiation effects on tumor cells. Amine functionalized Pt NFs allow facile surface chemistry and conjugation with various molecules, a major advantage to widen applications. In this study, Pt NFs were labeled with rhodamine so that their internalization can be tracked in vitro by confocal microscopy and FLIM. These NFs are efficiently uptaken by cells in cytoplasm, and subsequently enhance the radiation effect as demonstrated by clonogenic assays. It is shown that the induction of complex breaks plays a major role in the effect of NFs. These Pt NFs could be functionalized with ligands such as drugs, radionuclides or enzymes with the perspective to improve not only the efficiency of radiation treatments but also drug delivery and/or diagnostic performances.

## 4. Materials and Methods

### 4.1. Materials

Tetraammine platinum (II) chloride salt [Pt(NH_3_)_4_]Cl_2_⋅H_2_O, poly(ethylene glycol) diamine (H_2_N(CH_2_CH_2_O)nCH_2_CH_2_NH_2_, MW = 2000 g mol^−1^) and Rhodamine B isothiocyanate (RBITC) were purchased from Sigma-Aldrich (USA). Dulbecco’s Modified Eagle Medium (DMEM), fetal bovine serum (FBS), 0.25% Trypsin-EDTA, penicillin and streptomycin (100 U/mL), Hank’s Balanced Salt Solution (HBSS) and glutamine were purchased from Life Technologies (France). The pBR322 DNA plasmid (0.5 µg/μL) was purchased from Molecular Biology (France). Tris-EDTA (TE) buffer and Ethidium Bromide (EtBr, 1% solution) were purchased from Thermo Fisher Scientific (USA), Agarose D−5 was purchased from Euromedex (France). All chemical reagents were of analytical grade purity and used without further purification.

### 4.2. Preparation of Pt NFs

A highly versatile, eco-friendly and effective method based on water radiolysis was employed for the synthesis of Pt NFs as describe in the French Patent Application: Nanoparticules et procédé de preparation, FR1900008. Briefly, the reduction was achieved in aqueous solution using Tetraammine platinum (II) chloride salt [Pt(NH_3_)_4_]Cl_2_⋅H_2_O, as precursor and PEG-diamine as scavenger, stabilizer and capping agent. The sample solution was deareated by purging with N_2_ for 15 min and then irradiated with γ-rays using a panoramic Cobalt−60 source (E = 1.25 MeV, LET = 0.2 keV.μm^−1^) at a dose rate (determined by Fricke dosimetry) of 68.2 Gy.min^−1^. The optimised Pt NFs were synthesised with a PEG diamine: Pt molar ratio of ca. 50 and Pt ions were completely reduced to Pt^0^ in solution (10^−2^ mol L^−1^) at a dose of 100 kGy. The obtained Pt NFs were stored at 4 °C in the absence of light.

Pt NFs were transferred to cryovials and then were frozen to −80 °C overnight. The lyophilization was performed on a laboratory Alpha 1−2 LDplus lyophilizer (CHRIST, Germany) under vacuum (40 µBar) at −50 °C for 48 h. After 2 days, the vials were sealed and removed. Prior to use, MiliQ water (18.2 MΩ.cm at 25 °C) was added to the cryovials to achieve the concentration desired. Physicochemical properties of re-suspended Pt NFs remain constant.

### 4.3. Characterization of Pt NFs

#### 4.3.1. The Optical Absorption Measurements

The optical properties of Pt NFs were acquired with a CARY 300 Scan UV-Vis Spectrophotometer (Int. Agilent, USA) in the wavelength range of 200–800 nm (with an optical path length of 0.2 cm) at room temperature before and after irradiation. The samples were diluted to a final Pt concentration of 1 × 10^−3^ mol L^−1^ for UV–visible absorption measurements.

#### 4.3.2. Size, Shape and Charge Determination

The size and shape of the Pt NFs were evaluated by HR-TEM in a MET-STEM JEOL 1400 microscope operating at an accelerating voltage of 120 kV. Samples were prepared by drop casting onto carbon-formvar coated copper grids (200 mesh, Agar Scientific Ltd., UK) allowing natural drying prior observation. Manual measurements of about 2000 objects from 20-recorded images were converted to cumulative number-based size distribution histogram using the ImageJ 1.46r software. The hydrodynamic diameter was estimated by DLS (Cordouan Technologies, France) using the Sparse Bayesian Learning (SBL) algorithm. The Pt NFs surface charge, characterized by zeta potential (ζ-potential) was determined using the Smoluchowski approximation from a WALLIS zeta potential analyzer (Cordouan Technologies, France). All samples were prepared in Milli-Q water at a Pt concentration of 5 × 10^−4^ mol L^−1^.

#### 4.3.3. XPS

The electronic states and chemical composition of NFs were identified by XPS. The samples were prepared by drop casting on glass substrates and allowing drying in the air. XPS measurements were performed on a spectrometer (Thermofisher Scientific, USA) equipped with a monochromatic aluminum source (Al-Kα, hν = 1486.7 eV) using a spot of 400 µm and a hemispherical analyzer, at a take-off angle of 0°. Survey scans were acquired at a pass energy of 200 eV and a step of 1 eV while the narrow scans were acquired at a pass energy of 50 eV and a step of 0.1 eV. A “dual beam” flood gun was used for charge compensation. Data acquisition and interpretation were processed with the Thermo Avantage software, using a peak-fitting routine with Shirley background and symmetrical 70/30% Gaussian/Lorentzian peak shapes and a Doniach–Sunjic type function to fit the Pt metal asymmetric peaks. The homogeneity of samples was checked by measurement on up to 4 different points.

#### 4.3.4. FTIR

FTIR allowed determining the presence of –NH_2_ derivate functional groups and chemical interactions between Pt and PEG. The FTIR analysis was performed using the attenuated total reflection (ATR) module of an FTIR Spectrometer (Bruker Vertex 70). The transmittance FTIR spectra were recorded in the range of 400–4000 cm^−1^.

### 4.4. Cell Culture Conditions

The HeLa cancer cells (cervical cancer derived cells, ATCC CCL-2TM) were cultivated in DMEM supplemented with 10% FBS, 1% penicillin and streptomycin, 1% glutamine at 37 °C in a 5% CO_2_ air incubator in a humidified atmosphere.

### 4.5. Cytotoxicity Assessment

The cytotoxicity of Pt NFs was evaluated performing colony formation assays (CFA), which determine cells survival by measuring the ability of a single cell to proliferate and form a colony. Briefly, HeLa cells were seeded in T-25 ventilated culture flasks (BioLite 25 cm^2^ cell culture treated flask, Thermo Scientific) at a density of 2 × 10^5^ cells per flask. They were incubated 6 or 12h with enriched medium containing Pt NFs at three different Pt molar concentrations: 2.5 × 10^−4^, 5 × 10^−4^ and 10^−3^ mol L^−1^. HeLa cells incubated with an equal volume of ultrapure water were use as control. Following incubation, cells were harvested and re-seeded into petri dishes at different densities to yield approximately 100 surviving colonies per dish. After 2 weeks, the obtained colonies were fixed, stained and manually scored. The analysis of variance was performed using the ANOVAOneWay method provided by OriginPro8.5.

### 4.6. Fluorescent Labelling of Pt NFs

For dye labeling of Pt NFs, we used the isothiocyanate derivative of RBITC. Briefly, Pt NFs ([Pt] = 5 × 10^−3^ mol L^−1^) were mixed with RBITC (5 × 10^−4^ mol L^−1^). The mixture was stirred at room temperature for 24 h. Then the solution was dialyzed using Vivaspin20 (Sartorius Stedim Biotech, UK) to remove any free and nonspecifically adsorbed RBITC molecules. Dialysis has been performed until no RBITC could be detected in the dialysate by UV-vis spectroscopy. The RBITC functionalized Pt NFs were characterized by UV-Vis spectroscopy and fluorescence spectroscopy. Absorption spectra of Pt NFs, free RBITC and RBITC labeled Pt NFs were performed as described in the previous section (The optical absorption measurements). The fluorescence spectra of these samples were recorded at on a Fluorescence Spectrofluorometer (Cary Eclipse, USA) with a 10 mm path length quartz cuvette. Both excitation and emission slits were set at 5 nm, while the scan speed was maintained at 600 nm min^−1^. The samples were excited at 555 nm and the emission spectra were recorded in the wavelength range, 565−630 nm.

### 4.7. Pt NFs Localization and Quantification

The internalization of fluorescence labeled Pt NFs by HeLa cells was observed at Centre de Photonique BioMédicale using a Leica SP5 confocal laser-scanning microscope (CLSM) equipped with a live cell incubator and collected with a 63× high numerical aperture (1.4) oil immersion objective. HeLa cells were seeded in 8-well chamber (TPP, Switzerland) with glass slide at a density of 10,000 per well in 500 μL complete medium and incubated overnight. The next day, cells were incubated with Pt NFs functionalized with RBITC as a fluorescent marker at Pt concentration of 5 × 10^−4^ mol L^−1^ for 6 h, then rinsed twice with PBS and HBSS was added. Subsequently, the cells were subjected to live cell imaging using CLSM. RBITC was excited at 550 nm and the fluorescence emission was detected in the 570–670 nm range.

FLIM was performed on a Leica’s SP8 microscope at FALCON (for FAst Lifetime CONtrast) platform. Fluorescence signal was detected by exciting the fluorophores with pulsed lasers at wavelength of 550 nm and were detected using the internal hybrid detector in single-photon counting mode. At least 1000 photon events per pixel were collected in all cases (where each pixel equals 152 × 152 nm^2^) and the lifetime analysis was carried out using the Leica FALCON FLIM integrated software. On each FLIM images, at least three regions of interest (ROI) were selected. The acquired fluorescent decays were fitted with a bi-exponential decay. The fluorescence lifetime of RBITC alone and RBITC labeled Pt NFs were obtained by averaging the numerical values obtained in these regions.

ICP-MS was used to determine the exact quantity of Pt uptake by each HeLa cell. Cells were seeded at density of 1.5 × 10^6^ cells per flask and incubated for 24 h. The cells were then exposed to Pt NFs at a Pt concentration of 5 × 10^−4^ mol L^−1^ in complete medium for 6 h. The cells were washed with PBS, collected and stored at −80 °C. The quantification of Pt contained in cells using ICP-MS, was performed by the UT2A Company (Pau, France). In parallel, a cell culture grown without Pt NFs at the same cell density, was analyzed as a control.

### 4.8. Cell Irradiation with γ-Rays

In total, 2 × 10^5^ exponentially growing HeLa cells were plated in flask 12 h before irradiation. The cells were incubated with Pt NFs at a Pt concentration of 5 × 10^−4^ mol L^−1^, diluted in cell culture medium for 6 h. The cells were then irradiated by photon beam using a Cesium-137 (E = 0.66 MeV) irradiation system GSR-D1 at Institut Curie, France. After irradiation, cells were washed with PBS, trypsinized, counted and re-seeded into petri dishes at different densities to yield approximately 100 surviving colonies per dish. After 14 days, the colonies were fixed and stained with a 0.5% methylene blue-50% methanol solution. The colony counts were fit with a linear-quadratic model (LQM) by plotting the data on a log (% survival) vs. dose plot in Origin.

(4) $$\mathrm{SF}\left(D\right)=e^{-\left(\alpha D+{\beta D}^{2}\right)}$$

Here, SF is the cell surviving fraction, D the irradiation dose and α and β are parameters representing direct lethal and sub-lethal damage, respectively.

We quantified the effect of the NFs utilizing two methods commonly found in the literature. The sensitivity enhancement ratio at 2 Gy (SER_2Gy_) is the ratio of SF at 2 Gy for irradiation without and with Pt NFs. The DEF10% is the ratio of doses at 10% survival for irradiation without NFs versus with NFs.

### 4.9. Plasmid Damage Induced by Irradiation

The nanoscale effect of Pt NFs on the induction of single strand breaks (SSBs) and double strand breaks (DSBs) by Cobalt-60 γ-rays (E = 1.25 MeV, dose rate = 4.46 Gy/min) irradiation were evaluated according to the protocol of previous work [76]. Briefly, the as-synthesized Pt NFs were diluted in ultrapure water to reach a Pt concentration of 4.23 × 10^−5^ mol. The samples of plasmids–Pt NFs complexes, 6 aliquots (18 µL) each contained 1 µL of pBR322 plasmids (0.5 µg/μL), 12.3 µL of TE buffer, 2.4 µL of Pt NFs with additional pure water, were prepared and incubated for 1 h prior irradiation.

### 4.10. Statistical Analysis

All the experiments were performed with at least two replicas for each experimental or control group in each independent assay, and numerical data were reported as the mean ± SD. A two-tailed Student’s t-test was used for the statistical analysis and the value of *p* < 0.05 was considered statistically significant.

## 5. Patents

French Patent Application FR1900008 “Nanoparticules et procédé de préparation”.

## Figures and Tables

**Table 1:** Coefficients α and β, R^2^, Sensitizing Enhancement Ratio (SER) and Dose Enhancing Factor (DEF) for HeLa cells irradiated by γ-rays without/with the presence of Pt NFs.

Sample	α (Gy^−1^)	β (Gy^−2^)	R^2^	SER	DEF
Control	0.24 ± 0.01	0.049 ± 0.002	0.999	−	−
Pt NFs	0.37 ± 0.01	0.044 ± 0.003	0.999	23%	1.20

**Table 2:** Yields of controls and plasmids loaded with Pt NFs under γ-ray irradiation. The molecular Amplification Factor (mAF) is also reported.

Sample	Nanosize Damage Per Plasmid and Per Gy (×10^−5^)	mAF (%)
Control	28.1 ± 0.4	−
Pt NFs	53.1 ± 0.5	89.0 ± 4.1

## Supplementary Materials

Supplementary materials can be found at https://www.mdpi.com/1422-0067/21/5/1619/s1.

## Abbreviations

BEs	Binding Energies
DEF	Dose Enhancing Factor
DSBs	Doble Strand Breaks
EDTA	Ethylenediaminetetraacetic acid
EPR	Enhanced Permeability and Retention effect
FWHM	Full Width at Half Maximum
FTIR	Fourier Transform InfraRed
FLIM	Fluorescence Lifetime Imaging Microscopy
ICP-MS	Inductively Coupled Plasma Mass Spectrometry
LMCT	Ligand-to-Metal Charge Transfer
LQM	Linear Quadratic Model
mAF	Molecular Amplification Factors
MEF	Metal Enhanced Fluorescence
NREs	Nano-Radio-Enhancers
PBS	Phosphate Buffer Saline
PEG	Poly (ethylene glycol)
PEG-diamine	Poly (ethylene glycol) diamine
Pt NPs	Platinum Nanoparticles
RBITC	Rhodamine Isothiocyanate
SER	Sensitizing Enhancement Ratio
SSBs	Single Strand Breaks
SF	Surviving Fraction
XPS	X-ray Photoelectron Spectroscopy
