# Supplementary material for: A Facile One-Pot Synthesis of Versatile PEGylated Platinum Nanoflowers and Their Application in Radiation Therapy
Source: Int J Mol Sci. 2020 Feb 27;21(5):1619. doi: 10.3390/ijms21051619 (PMC7084439; doi:10.3390/ijms21051619)
Supplement: Supplementary file 1 [file ijms-21-01619-s001.pdf]

Supplementary information

A Facile One-Pot Synthesis of Versatile PEGylated Platinum Nanoflowers and Their Application in Radiation Therapy

Xiaomin Yang, Daniela Salado-Leza, Erika Porcel, César R. González Vargas, Farah Savina, Diana Dragoe, Hynd Remita, Sandrine Lacombe

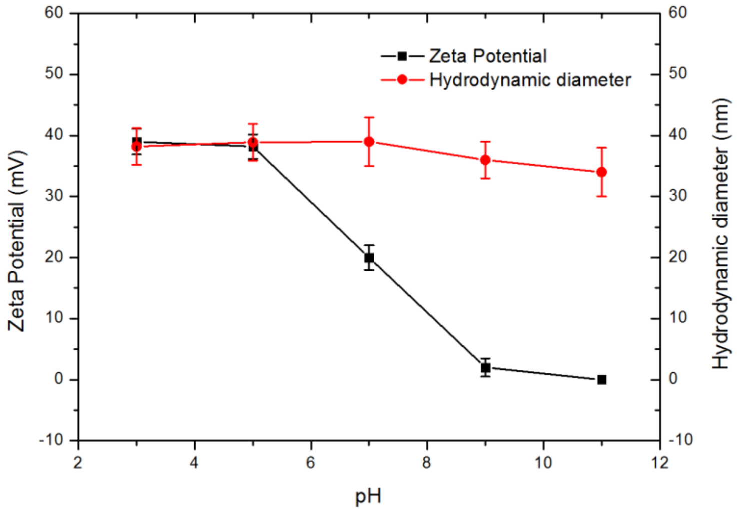

Figure 1. The hydrodynamic diameter and zeta potential of Pt NFs in aqueous solution at various pH from 3 to 11, by adding 0.1 M NaOH and HCl to adjust pH.

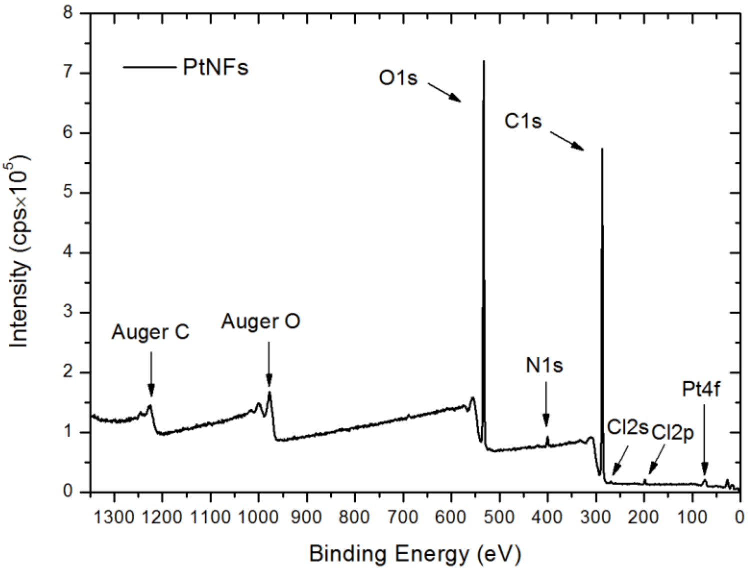

Figure 2. X-ray Photoelectron Spectrometry (XPS) survey spectrum of Pt NFs.

Table S1. XPS data summary: Binding Energy (BE), assignment and FWHM of Pt NFs.

Sample	Atomic core level	BE (eV)	Assignment	FWHM (eV)
Pt NFs	Pt-4f7/2 surf.	70.8	Pt0 surf.	1.4
	Pt-4f5/2 surf.	74.1	Pt0 surf.	1.4
	Pt-4f7/2 core	72.8	Pt0 core	2.0
	Pt-4f5/2 core	76.1	Pt0 core	2.0
	C-1s	286.8	C-O & C-N	1.2
	C-1s	285.3	C-H & C-C	1.2
	O-1s	533.2	C-O-H & C-O-C	1.3
	N-1s	402.0	C-NH3+	1.6
	N-1s	400.6	C-NH2	1.5
	N-1s	399.5	C-N & C=N	1.4

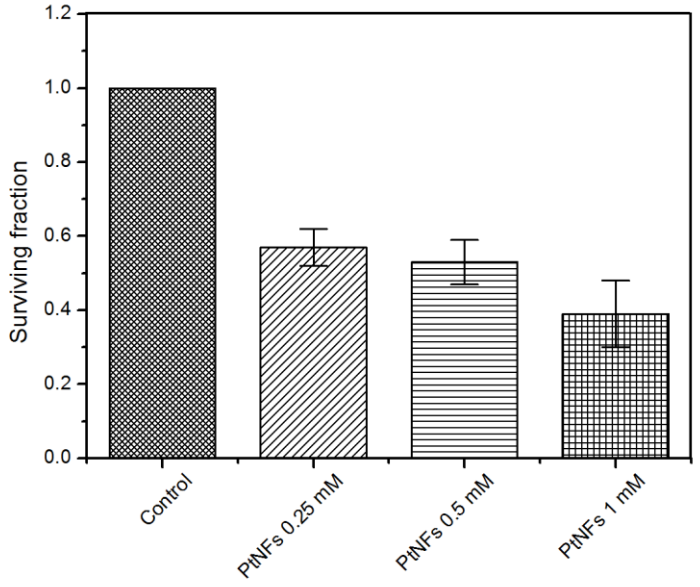

Figure 3. Cytotoxicity determined by clonogenic assay following a 12 h exposure to medium containing Pt NFs at different Pt concentrations of 2.5×10^{-4}, 5×10^{-4} and 10^{-3} $mol\cdot l^{-1}$. Data represented are mean ±SD of three identical experiments made in triplicate.

Detailed calculation of the lifetime

Fluorescence lifetime imaging microscopy (FLIM) has become a powerful imaging tool in cell and molecular biology research. Fluorescence lifetime can be defined as the average time fluorophores stay in the excited state after excitation and is independent of fluorescent intensity [1]. In lifetime measurement, a short-pulsed laser is used to excite the fluorophores and the fluorescent emission is measured as a function of intensity decay over time. Our results show the bi-exponential decay of RBITC and RBITC labeled Pt NFs,

$$I = A_1 e^{-(t/\tau_1)} + A_2 e^{-(t/\tau_2)} \quad (1)$$

Where A_1 and A_2 are the relative contribution of the individual decays with lifetime τ1 and τ2, respectively. The criteria for an acceptable fit were ascribed by Zanello M. [2]: 1) a χ2 value less than 1.0 and 2) residuals randomly distributed around 0 within the interval +4 and–4.

Afterwards, the average weighted lifetime τ_0 are calculated according to reference [3]. The values of all the parameters to obtain fluorescence lifetime are presented in table 2.

$$\tau_0 = f_1\tau_1 + f_2\tau_2 \quad (2)$$

$$f_1 = \frac{A_1}{A_1+A_2} \quad (3)$$

$$f_2 = \frac{A_2}{A_1+A_2} \quad (4)$$

Where f_1 and f_2 are fractional contributions, τ_1 and τ_2 are individual lifetimes of each component.

Table S2. Experimental data of lifetime measurements of RBITC and RBITC labeled Pt NFs. f_1, f_2 are fractional contributions, τ_1, τ_2 are lifetimes of two exponents, τ_0 is the average lifetime.

**Sample**	f_1	f_2	τ_1 (ns)	τ_2 (ns)	τ_0 (ns)
RBITC	0.50	0.50	1.6	3.4	2.5
RBITC labeled Pt NFs	0.24	0.76	1.2	2.7	2.3

References

1. Leung, R. W. K.; Yeh, S.-C. A.; Fang, Q., Effects of incomplete decay in fluorescence lifetime estimation. Biomedical optics express 2011, 2, (9), 2517-2531.
2. Zanello, M.; Poulon, F.; Varlet, P.; Chretien, F.; Andreiuolo, F.; Pages, M.; Ibrahim, A.; Pallud, J.; Dezamis, E.; Abi-Lahoud, G., Multimodal optical analysis of meningioma and comparison with histopathology. J Biophotonics 2017, 10, (2), 253-263.
3. Salas Redondo, C.; Kleine, P.; Roszeitis, K.; Achenbach, T.; Kroll, M.; Thomschke, M.; Reineke, S., Interplay of fluorescence and phosphorescence in organic biluminescent emitters. The Journal of Physical Chemistry C 2017, 121, (27), 14946-14953.
